# Palmitate induces DNA damage and senescence in human adipocytes *in vitro* that can be alleviated by oleic acid but not inorganic nitrate

**DOI:** 10.1016/j.exger.2022.111798

**Published:** 2022-04-04

**Authors:** Abbas Ishaq, Tamara Tchkonia, James L. Kirkland, Mario Siervo, Gabriele Saretzki

**Affiliations:** a Biosciences Institute, Campus for Ageing and Vitality, Newcastle upon Tyne, UK; b Department of Physiology and Biomedical Engineering, Mayo Clinic, Rochester, United States of America; c Human Nutrition Research Centre, Population Health Sciences Institute, Newcastle University, Newcastle upon Tyne, UK; d School of Life Sciences, University of Nottingham, Queen’s Medical Centre, Nottingham, UK

**Keywords:** Human adipocytes, Cell culture, Senescence, DNA damage, Palmitate, Nitrate, Oleic acid

## Abstract

Hypertrophy in white adipose tissue (WAT) can result in sustained systemic inflammation, hyperlipidaemia, insulin resistance, and onset of senescence in adipocytes. Inflammation and hypertrophy can be induced *in vitro* using palmitic acid (PA). WAT adipocytes have innately low β-oxidation capacity, while inorganic nitrate can promote a beiging phenotype, with promotion of β-oxidation when cells are exposed to nitrate during differentiation.

We hypothesized that treatment of human adipocytes with PA *in vitro* can induce senescence, which might be attenuated by nitrate treatment through stimulation of β-oxidation to remove accumulated lipids. Differentiated subcutaneous and omental adipocytes were treated with PA and nitrate and senescence markers were analyzed.

PA induced DNA damage and increased p16^INK4a^ levels in both human subcutaneous and omental adipocytes *in vitro*. However, lipid accumulation and lipid droplet size increased after PA treatment only in subcutaneous adipocytes. Thus, hypertrophy and senescence seem not to be causally associated. Contrary to our expectations, subsequent treatment of PA-induced adipocytes with nitrate did not attenuate PA-induced lipid accumulation or senescence. Instead, we found a significantly beneficial effect of oleic acid (OA) on human subcutaneous adipocytes when applied together with PA, which reduced the DNA damage caused by PA treatment.

## 1. Introduction

Ageing and obesity are two important health risks for people worldwide. Overfeeding results in storage of excess energy as triglyceride lipid droplets in adipocytes of white adipose tissue (Laviola et al., 2006). Hypertrophic adipocytes, particularly in visceral adipose tissue depots, initiate pro-inflammatory responses in local adipose tissue, which, if sustained, can lead to systemic inflammation, hyperlipidaemia, and insulin resistance (Laviola et al., 2006; Osborn and Olefsky, 2012; Del Corno et al., 2016). However, this inflammation reaction in adipose tissue might depend on the presence of macrophages that are missing from cultured adipocytes.

In healthy adipocytes, lipid content is regulated by lipolysis and controlled release of free fatty acids (FFA) into the bloodstream (Laviola et al., 2006; Ahmadian et al., 2010; Ahmadian et al., 2007; Ahmadian et al., 2010; Walther and Farese, 2012). Fatty acids (FA) are utilized as an energy source through mitochondrial β-oxidation in other energy demanding organs, such as cardiac and skeletal muscles, liver, or kidney (Houten and Wanders, 2010). Characteristically, adipocytes of white adipose tissue possess low β-oxidation activity, unlike those in thermogenic brown adipose tissue (Frayn et al., 2008; Lee et al., 2015). The term “beiging” refers to an increase in abundance of brown relative to white adipocytes, either by conversion of white adipocytes into brown or by a switch in differentiation of pre-adipocytes from white into brown adipocytes (Servera et al., 2014; Rosell et al., 2014; Lu et al., 2012; Lo Kinyui and Sun, 2013; Thyagarajan and Foster, 2017). Nevertheless, brown and white adipocytes are generally distinct subtypes of cells.

Excess dietary fat intake has been associated with increased risk of weight gain and fat deposition in mice and humans and poses a serious health risk (Hu et al., 2018; Hill et al., 2000). FA are classified as saturated or unsaturated depending on the presence or absence of double bonds, respectively. Mono- and polyunsaturated FA such as oleic or linoleic acid have been linked to protective effects against several adverse health outcomes, whereas saturated FA such as palmitic or myristic acid are generally considered detrimental (Palomer et al., 2018; Maedler et al., 2001; Maedler et al., 2003). Oleic acid (OA) is abundant in olive oil, which represents a key element responsible for the established, beneficial effects of the Mediterranean diet. Palmitic acid (PA) is part of meat and dairy products and has been shown to increase LDL (low density lipoprotein) cholesterol (Kien et al., 2014). Replacing dietary PA with OA increases insulin sensitivity in humans (Vessby et al., 2001), reduces the impact of higher cholesterol intake and risk of cardiovascular disease (Kien et al., 2014), and can alleviate detrimental cellular effects of PA, such as release of cytochrome *c* and the associated Bcl-2-associated apoptosis in human β-cells (Maedler et al., 2003). PA can disrupt endoplasmic reticulum integrity by increasing saturated lipid synthesis (Borradaile et al., 2006; Leamy et al., 2014; Robblee et al., 2016; Shen et al., 2017) and activating pro-inflammatory pathways through Toll-like receptor 4 (TLR4). This is similar to effects due to the Lipid A component of inflammatory lipo-polysaccharide (LPS) in Chinese hamster ovary (CHO) cells, rat hepatoma cells (Velloso et al., 2015), RAW 264.7 murine macrophage-like cells (Lee et al., 2001), and 3T3-L1 mouse pre-adipocytes (Schaeffler et al., 2009). PA treatment can also increase endoplasmic reticulum stress, inflammation, and apoptosis in adipocytes *in vitro* (Permana et al., 2006; Suganami et al., 2005; Guo et al., 2007; Suganami et al., 2007; Takahashi et al., 2008; Kennedy et al., 2009).

Senescence in dividing cells is characterized by an essentially irreversible arrest of cell division. It is also characterized by increase in general and telomere-associated DNA damage, upregulation of *p21*^*CIP1*^ and *p16^INK4a^* expression, p53 activation, mitochondrial biogenesis and dysfunction, as well as the senescence-associated secretory phenotype (SASP) that can include production of pro-inflammatory cytokines (Lopez-Otin et al., 2013; Passos et al., 2010). Post-mitotic cell types under stress conditions can also develop these markers in a form of cell cycle-independent senescence (Jurk et al., 2012; Ogrodnik et al., 2017; Ishaq et al., 2018a; Ishaq et al., 2018b). Within adipose tissue, clearance of p16^INK4a^-positive pre-adipocytes can prevent loss of fat mass due to ageing (Xu et al., 2015). Linking senescence during metabolic stress to lipid accumulation in adipose tissue has emphasized the potential importance of adipocytes during metabolic stress (Sepe et al., 2010). However, most studies so far have analyzed ageing and senescence in pre-adipocytes rather than in differentiated adipocytes (Tchkonia et al., 2010).

Our previous *in vivo* studies in mouse visceral adipose tissue showed that ageing and *ad libitum* feeding caused adipocyte senescence, which could partially be alleviated by dietary restriction (Ishaq et al., 2018a; Ishaq et al., 2018b). Compared to subcutaneous adipocytes, visceral adipocytes reportedly have increased susceptibility to changes in signaling molecules such as catecholamines and insulin (Ostman et al., 1979; Bolinder et al., 1983). Visceral WAT volume is associated with the development of glucose intolerance and type 2 diabetes (Ostman et al., 1979; Bolinder et al., 1983; Zhu et al., 2015), as well as incidence of colon (Oh et al., 2008) and breast cancer (Schapira et al., 1994). The visceral (omental) pre-adipocyte secretome is more predisposed to recruit macrophages and induce an inflammatory phenotype in them compared to subcutaneous pre-adipocytes (Zhu et al., 2015). Abdominal subcutaneous adipocytes, on the other hand, seem to have higher adipogenic potential than omental adipocytes, proliferate faster, and are more resistant to apoptosis (Tchkonia et al., 2005). In this study, we used the size of lipid droplets as a proxy-read-out marker of obesity and also show its tight association to markers of senescence.

Nitric oxide (NO) is a gaseous signaling molecule that is involved in several physiological functions, including control of vasomotor tone, immunity, coagulation, energy metabolism, and neurotransmission (Yetik-Anacak and Catravas, 2006). Deficiency of NO has been linked to loss of endothelial integrity and is involved in the pathogenesis of atherosclerosis, insulin resistance, and mitochondrial dysfunction (Yetik-Anacak and Catravas, 2006). Reduced production of whole-body NO has been observed in patients with hypertension, metabolic syndrome, and diabetes (Martyn et al., 2008; Siervo et al., 2011). Supplementation of young and older obese individuals with a standardized dose of inorganic nitrate after induction of hyperglycaemia reduced the level of reactive oxidative species (ROS) in circulating peripheral blood mononuclear cells in the older cohort (Ashor et al., 2016). Furthermore, nitrate supplementation during differentiation of rat primary pre-adipocytes promoted beiging in differentiating adipocytes (Roberts et al., 2015). Nitrate is reduced to nitrite and NO in cell culture in a low-oxygen environment by xanthine oxidoreductase, upregulating cytosolic cGMP and protein kinase G (Roberts et al., 2015). This, in turn, upregulates mitochondrial biogenesis through peroxisome proliferator-activated receptor γ coactivator 1-α (PGC-1α) as well as carnitine palmitoyl-transferase 1 (CPT1) and uncoupling protein 1 (UCP1), an electron transport chain uncoupler mainly expressed in brown adipose tissue (Roberts et al., 2015; Wu et al., 1999). The concept of increasing mitochondrial uncoupling in adipocytes to increase β-oxidation is not novel. There is a vast body of studies on the induction of beiging through small molecules such as berberine, butein, and β3-AR agonists (Zhang et al., 2014; Song et al., 2016; van Dam et al., 2015; Maeda et al., 2005; Ohno et al., 2012; Song et al., 2017). In previous studies, attempts have been made to promote mitochondrial biogenesis and induce beiging by upregulating PGC-1α levels (Frayn et al., 2008; Maassen et al., 2007; Mazzucotelli et al., 2007). However, increasing only PGC-1α by transducing a PGC1-α-carrying adenovirus into primary human subcutaneous adipocytes, followed by activation using PPARα and PPARγ agonists, was insufficient to enhance β-oxidation capability (Frayn et al., 2008; Maassen et al., 2007; Mazzucotelli et al., 2007), while exposing primary adipocytes to inorganic nitrate increased fatty acid uptake and β-oxidation (Roberts et al., 2015).

To our knowledge, there are no direct studies that have analyzed senescence in cultured human adipocytes exposed to increased lipid stress from PA. We hypothesized that lipid accumulation due to saturated FA might cause adipocyte senescence. We found that a hypertrophic phenotype induced by treatment of human subcutaneous adipocytes *in vitro* with PA resulted in upregulation of senescence markers including γH2A.X DNA damage foci, telomere-associated foci (TAF), and p16^INK4a^ levels. We also hypothesized that this senescence and the associated markers might be alleviated by inorganic nitrate. However, we were unable to find beneficial effects of nitrate treatment after PA incubation *in vitro*. Instead, we found that simultaneous treatment of cultured adipocytes with PA and OA prevented the occurrence of senescence-associated and inflammatory markers. We also detected differences in this regard between human subcutaneous and omental adipocytes.

## 2. Materials and methods

### 2.1. Tissue culture: differentiation and treatment of human primary adipocytes

2.1.1. Primary human subcutaneous pre-adipocytes (Poietics™ Subcutaneous Pre-adipocyte Cell System) were obtained from Lonza/Amaxa, expanded, and grown at 20% O_2_ in PBM-2 medium (Lonza) according to the Lonza protocol.

2.1.2. White adipose tissue as the source for pre-adipocytes was obtained during abdominal surgery of 5 healthy kidney donors following informed written consent at Mayo Clinic (Rochester, MA, USA). Ethical approval for the procedure was granted by the Mayo Clinic Institutional Review Board. Donors for the study were females with a mean age of 41.4 ± 4 (SEM) years and mean body mass index (BMI) of 28.56 ± 1.47 (SEM) kg/m^2^. Abdominal subcutaneous (outside the *fascia superficialis*) and greater omental WAT were obtained from subjects in parallel as described earlier (Tchkonia et al., 2002). Five pairs of isogenic donor-derived human subcutaneous and visceral/ omental pre-adipocytes isolated and initially cultivated at Mayo Clinic were used in our study at passage 4.

**Fig. 1.**
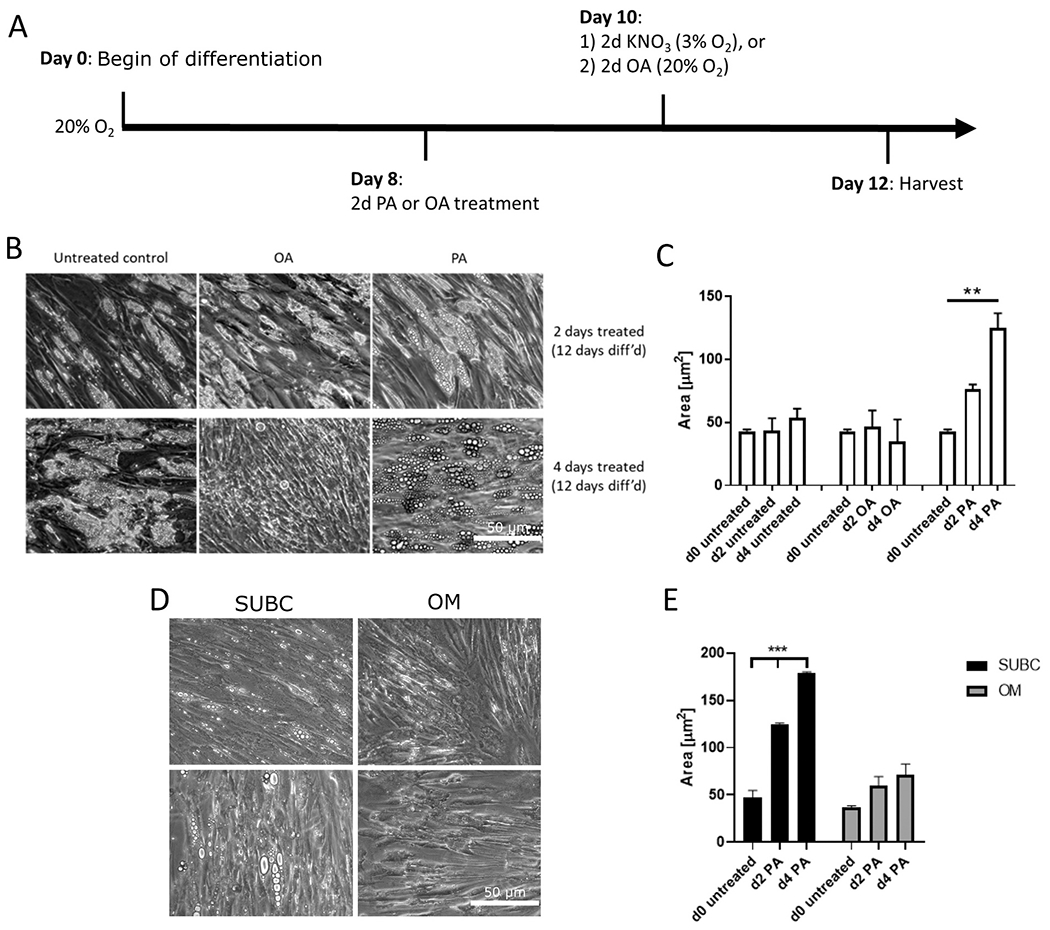
Effect of 2 and 4-day FFA treatments on lipid accumulation in human primary subcutaneous adipocytes. Human primary adipocytes were differentiated for 8 days, then treated with 150 nM of OA or PA for 4 days. A) Schematic diagram for differentiation and treatment of the human primary adipocytes. B) Representative images (200× magnification, scale bar is 50 μm) of lipid droplet sizes in subcutaneous adipocytes (Lonza) after days 2 and 4 of treatment. C) Change in lipid droplet size in subcutaneous adipocytes (Lonza), quantified as area (μm^2^). D) Representative images (200× magnification, scale bar is 50 μm) of lipid droplet sizes in donor-derived subcutaneous (SUBC) and omental (OM) adipocytes at day 2 of treatment. Upper and lower rows are from different individual donors. E) Change in lipid droplet size in donor-derived subcutaneous and omental adipocytes after FFA treatment, quantified as area (μm^2^). ** p < 0.01, *** p < 0.001 by one-way ANOVA and Holm-Sidak *post hoc* test.

Differentiation and analyses of both adipocyte types were performed pairwise. They were expanded in individual wells in 6well plates in PBM-2 medium until 90% confluent. The cells were dissociated using TrypLE (Gibco) for 3 min at 37 °C. The pre-adipocytes were seeded onto 19 mm glass coverslips in 12-well plates at a density of 20,000 cells per well. After 4–6 days, at 100% confluence, PBM-2 differentiation medium was added to the pre-adipocytes according to the Lonza protocol. At day 8 of differentiation, palmitic or oleic acid (150 nM after FFA-free BSA conjugation (Sigma-Aldrich)) were added to the appropriate wells. At day 10 of differentiation, KNO_3_ (100 μM) was added to the appropriate wells (“after”-differentiation). After addition of KNO_3_, cells were incubated in 3% O_2_ to allow for xanthine oxidase/ reductase-based nitrate reduction until day 12. The time course of treatment is depicted in Fig. 1A. The postmitotic status of the differentiated adipocytes was validated using a Ki67 antibody (Abcam 92742).

The differentiated and treated adipocytes were then prepared for downstream analysis. Brightfield images were taken at 200× magnification at days 8, 10, and 12 for lipid droplet size quantification. Automated quantification of lipid droplet sizes (area, μm) of each image was performed using a protocol in the Icy image analysis software 1.9.4.1 (de Chaumont et al., 2012) (http://icy.bioimageanalysis.org/).

2.1.3. MRC-5 human lung fibroblasts (EACC, London, UK) were expanded and grown at 20% O^2^ in DMEM (Gibco) with 10% FBS. The cells were dissociated using TrypLE (Gibco) for 3 min at 37 °C. 20,000 cells per well were seeded onto 19 mm (Osborn and Olefsky, 2012) coverslips in 12-well plates. After 24 h, the cells were x-irradiated (20 Gy) at 250 kV, 13.3 mA for 10 min in a x-Rad 225/CT10 (Omyxis, Poland). Medium was changed immediately after irradiation and the cells were incubated for a further 10 days for development of a p16^Ink4a^-positive senescence phenotype. The non-irradiated controls were fixed at day 0 of irradiation. Hydrogen peroxide (H_2_O_2_) treatment was performed at 150 μm H_2_O_2_ (SIGMA) in serum-free DMEM for 2 h. After that treatment was stopped with DMEM containing 10% FCS and cells fixed immediately with 4% PFA in PBS.

### 2.2. Measurement of nitrate and nitrite concentrations

Residual nitrate and nitrite concentrations in the growth media after KNO_3_ treatments were measured to evaluate internalization by adipocytes. The nitrate-containing media were first deproteinated by adding two volumes of cold 70% ethanol to the samples, which were then vortexed for 10 s and incubated on ice for 30 min. The samples were then centrifuged at 3220 ×*g* for 5 min. The supernatants were then placed in clean 1.5 ml centrifuge tubes and nitrate and nitrite concentrations were measured. Measurements of nitrate and nitrite concentrations were performed using ozone-based chemiluminescence with a NO analyser (Sievers 280i, Analytix, UK), as previously described (Shannon et al., 2016). Nitrite concentrations were determined by adding the samples to sodium iodide (0.17 M) in glacial acetic acid under nitrogen at room temperature. Nitrate concentrations were determined by adding the samples to vanadium chloride (0.1 M) in hydrochloric acid (1 M) at 95 °C.

### 2.3. Immunofluorescence staining and analysis

After treatment, cells on coverslips were fixed with 4 % paraformaldehyde for 10 min. 1 ml of PBS was added per well and the plates were stored at 4 °C until staining. All following PBS washes were performed thrice for 5 min unless otherwise indicated. For immunofluorescence staining, the cells were blocked with 1% normal goat serum (NGS) in bovine serum albumin (BSA) for 30 min. The samples were then sequentially blocked with avidin and biotin for 15 min each with PBS washes after each step (SP-2001, Vector Lab). After aspiration, rabbit monoclonal primary antibody to γH2A.X (1:500, 9718, Cell Signaling Technologies) in NGS blocking solution was added to each well for 1 h at room temperature on a rocking table. The samples were then incubated with 1:500 of biotinylated anti-rabbit IgG antibody (PK-4002 Vectastain ABC kit, Vector labs), followed by 1:1000 of fluorescein-labelled avidin DCS (A-2011-1, Vector labs) for 1 h at room temperature on a rocking table. After aspiration and rinsing with PBS, primary antibody to p16^INK4a^ (1:50, SC-468, Santa Cruz; 1:5, CINtec Histology kit 9511) followed by 1:1000 of fluorescently labelled goat anti-rabbit secondary antibody (AlexaFluor 488; Molecular Probes) in NGS blocking solution were added to the samples for 1 h at room temperature with PBS washes in between. After another PBS wash, DAPI (Cystain UV ploidy, Sysmex) was added to the samples for 10 min at room temperature. The samples were then mounted in 7 μl of Vectashield antifade mounting medium (Vector Labs). For co-localisation of γH2A.X with telomeres, TAFs, a γH2A.X staining protocol, using fluorescein-labelled avidin DCS hybridization, was performed. The samples were then fixed with 4% paraformaldehyde for 10 min and sequentially dehydrated in 70%, 90%, and 100% ethanol for 3 min per step. 15 μl of Cy3-labelled CCCTAA telomere PNA probe (PanaGene, South Korea) in hybridization buffer (2.5 μg/ml PNA probe, 70% deionized formamide (Sigma-Aldrich), 25 mM MgCl_2_, 5% Roche blocking buffer (2 μl of 10× stock Roche blocking reagent in 18 μl of autoclaved maleic acid at pH 7.5, Roche), 2.5 μl Tris buffer (1 M, pH 7.2, Sigma-Aldrich), and deionized H_2_O) were applied to the coverslips, which were inverted onto a glass dish to ensure full liquid contact for 10 min at 80 °C, followed by 2 h at room temperature as described previously (Hallam et al., 2015). The samples were then washed with 70% formamide (Sigma-Aldrich) in 2× SSC buffer, DAPI (Cystain UV ploidy, Sysmex) was applied for 10 min, and the samples were mounted onto glass slides in Vectashield antifade mounting medium (H-1000, Vector Lab). For each sample, 7 fields of z-stack images (0.45 μM per stack) were acquired at 400× magnification using a Leica DMi8 microscope (Leica Microsystems).

For each sample, 30 images were taken over 6 × 5 fields of view at 2080 × 2080 pixels each using a Leica DMi8 microscope (Leica Microsystems). Seven images were randomly selected and analyzed for each group per experiment.

TAF and γH2A.X nuclear focus counts as well as nuclear p16^INK4a^ intensities were quantified using automated batch-analyses protocols in Icy version 1.9.4.1 (de Chaumont et al., 2012). An untreated sample was used as a negative control for nuclear staining to determine an intensity threshold below which p16^INK4a^ signals were considered negative. This was done in order to account for perinuclear staining below the plane of focus and other background fluorescence. The nuclear p16^INK4a^ intensities were then thresholded and summarized in Excel, where p16^INK4a^ intensities above the threshold were counted as p16^INK4a^ positive.

### 2.4. RNA extraction, reverse transcription, and qPCR

For each treatment, a sample from one well of a 12-well plate was homogenized by vigorously pipetting with 500 μl of Qiazol (Qiagen). RNA isolation, reverse transcription, and qPCR were performed using a Qiagen RNeasy kit as previously described (Ishaq et al., 2018b). 18S primers were used as the housekeeping gene. Values were expressed as a 2^−ΔΔCt^ average for each sample. Primer sequences are given in Table 1.

### 2.5. Mitochondrial superoxide measurements

Cells were trypsinized and resuspended in 4 ml of serum-free medium containing 5 μM of MitoSOX (Invitrogen) and incubated for 30 min at 37 °C. MitoSOX intensity was measured on a flow cytometer (Partec, Muenster, Germany). Cell population was selected and gated using unstained controls which also served as background levels which were subtracted from MitoSox stained samples. Up to 10,000 counts were used to determine mean forward scatter for each treatment. Data are shown as fold-change vs. untreated control.

### 2.6. Statistical analyses

Statistical analysis was performed using SigmaPlot 12.5 (Systat Software Inc., USA). All data sets were tested for normal distribution using Shapiro-Wilks test, then analyzed by One Way ANOVA. Holm-Sidak *post hoc* test was used for pairwise comparison of different groups, *t*-tests were performed where appropriate (for comparing two groups). For multiple comparisons, p-values from *post hoc* tests at p < 0.05 were considered significant. Graphs were generated using GraphPad Prism 8 (GraphPad Software, USA). All bars show means and SEM of samples from either 3 independent differentiation experiments of Lonza-adipocytes or 3 independent donors of Mayo-derived adipocytes. * p < 0.05, ** p < 0.01, ***p < 0.001.

## 3. Results

### 3.1. Differential effects of palmitic (PA) and oleic acid (OA) on lipid accumulation in human subcutaneous adipocytes

Differentiation of human subcutaneous pre-adipocytes (Lonza) over the course of 8 days resulted in an increase in lipid droplet (LD) accumulation, which is a measure for hypertrophy (Fig. 1B, D). Treatment of cultured human subcutaneous adipocytes (Lonza) with PA increased lipid droplet size from 42.9 ± 1.5 μm to 76.0 ± 3.7 μm after two days (days 8–10) (p = 0.017), and to 124.6 ± 11.8 μm after four days (days 10–12) (p < 0.001) (Fig. 1C). Basal lipid droplet size in the absence of FFA continued to increase between days 8 and 12, from 42.9 ± 1.5 μm to 53.4 ± 7.4 μm. Thus, background lipid accumulation only accounted for 13% of the lipid accumulation after 4 days of PA treatment. Intriguingly, adipocytes treated with OA showed no difference to the untreated control (Fig. 1B, middle panel). Thus, while PA treatment promoted an accelerated increase in lipid droplet size in human subcutaneous adipocytes, OA did not induce any measurable change in lipid droplet size over 4 days.

Next, we examined whether our findings in human adipocytes from Lonza could be replicated across multiple donors. In addition, we were interested in whether there might be any differences in response to PA between subcutaneous and visceral/ omental adipocytes. This change in LD size was reflected in PA treatment of the donated (Mayo) human subcutaneous adipocytes after the same differentiation conditions (Fig. 1D, E). LD size at day 8 was strikingly similar to that in the Lonza-derived adipocytes at day 8. Interestingly, the Mayo-derived subcutaneous adipocytes accumulated far more lipid after 2 and 4 days of PA treatment than omental, resulting in LD sizes of 125.1 ± 0.95 μm at day 2 and 179.4 ± 0.87 μm at day 4 (p < 0.001, Fig. 1E). The omental adipocytes showed a trend for LD size increase after PA treatment that did not reach statistical significance. These data confirm that subcutaneous adipocytes are more responsive to lipid accumulation induced by PA than omental adipocytes.

### 3.2. Palmitic acid induces DNA damage and increase in p16^INK4a^ staining without increase in the expression of inflammatory markers or a significant increase of superoxide

**Fig. 2.**
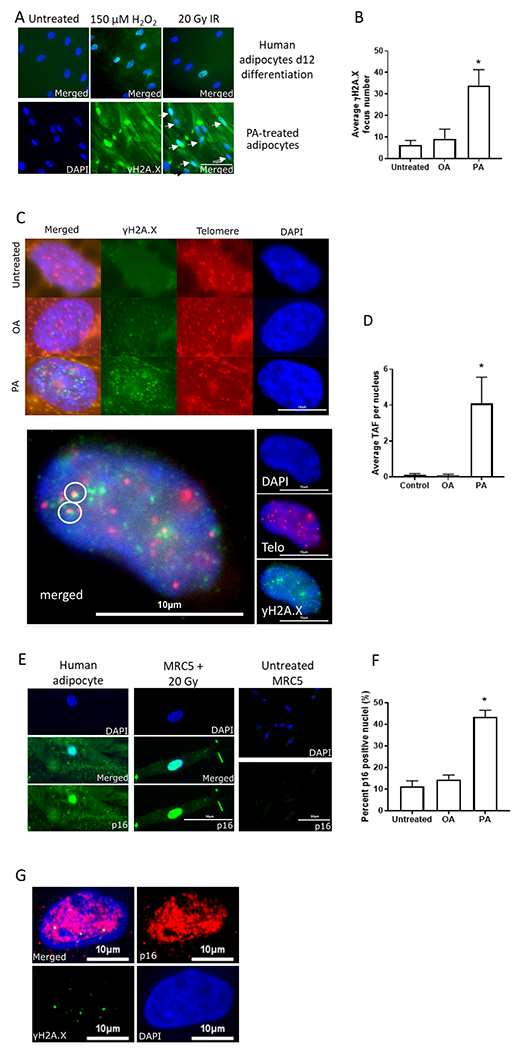
Effect of 4 days 150 nM FFA treatment on senescence markers in human primary subcutaneous adipocytes (Lonza). A) Comparison of γH2A.X DNA damage foci in controls after 150 μM H_2_O_2_ and 20 Gy x-irradiation (IR) (upper row, merged images), and PA treatment in differentiated (12 days) human adipocytes (lower row, single plus merged image) (400×, scale bar is 50 μm). B) Average γH2A.X DNA damage focus counts per nucleus after FFA treatment. C) Upper row: representative images from control and OA- and PA- treated adipocytes. Blue: DAPI nuclear staining, green: γH2A.X, red: telomere probe (400×, scale bar is 10 μm). In the left column are the merged ones. Lower row: An enlarged image of a nucleus with TAFs. Small insets on the right: DAPI staining (blue), telomere probe (red), and γH2A.X signal (green). (400×). Large image on the left shows co-localization of DNA damage and telomeres in white circles. D) Average telomere-associated focus count per nucleus after FFA treatment. E) Representative images of p16^INK4a^ IF staining in MRC-5 fibroblasts10 days after 20 Gy x-IR (middle row, scale bar is 30 μm) and human primary subcutaneous adipocyte after 4 days of PA treatment (left row) as well as a negative control of untreated MRC-5 on the right at 400× magnification (scale bar is 50 μm) (Top: DAPI only, Middle: Merged, Bottom: p16^INK4a^ only. For untreated MRC5 cells, only DAPI (top) and p16^INK4a^ (bottom) are provided. F) Percentage of p16^INK4a^ -positive nuclei quantified by immunofluorescence staining after FFA treatment. G) Double IF staining for p16^INK4a^ (red) and γH2A. X (green) of a PA-treated adipocyte (400×, scale bar is 10 μm). * p < 0.05 by one-way ANOVA and Holm-Sidak *post hoc* test. (For interpretation of the references to colour in this figure legend, the reader is referred to the web version of this article.)

Fig. 2A shows representative images of human adipocytes treated with H_2_O_2_ and x-irradiation (upper row) as positive controls, together with PA treated (150 nM) adipocytes (lower row) using a γH2A.X antibody for visualizing DNA damage foci. Treatment of cultured and differentiated human subcutaneous adipocytes (Lonza) with PA for 4 days (days 8 to 12) increased mean γH2A.X DNA damage focus count by 5.5-fold from 6.2 ± 2.2 to 34.0 ± 7.3 foci per nucleus (p = 0.025) (Fig. 2B).

In addition to general genomic DNA damage, we determined the mean number of telomere-associated foci (TAF), which increased from 0.1 ± 0.1 to 4.1 ± 1.5 foci per nucleus (p = 0.031) (Fig. 2C, D). In contrast, mean number of γH2A.X foci and TAF counts did not significantly change with OA treatment at the same time point (Fig. 2B, D). Fig. 2E shows a representative p16^INK4a^ image of MRC-5 fibroblasts after x-irradiation (middle panel) as well adipocytes treated with 150 nM PA for 4 days (left panel). The right panel shows untreated MRC-5 cells. Treatment of cultured human subcutaneous adipocytes with PA increased the number of p16^INK4a^ positive nuclei 4-fold (p < 0.05), from 11.2 ± 2.6 to 43.4 ± 3.1 p16^INK4a^ -positive nuclei (Fig. 2F). Similarly to general DNA damage and TAFs, OA treatment did not induce an increase in p16 positive staining in adipocytes.

In order to validate the senescent phenotype, we performed a double IF staining for p16^INK4a^ and γH2A.X, which both co-exist in the nuclei of PA-treated adipocytes (Fig. 2G).

Surprisingly, despite the increase in the staining frequency of p16^INK4a^ positive cells, after PA treatment, expression of p16 did not increase. There was only a slight increase in the expression of p21^Cip1^ after PA treatment (suppl Fig. 1A) while sen-beta gal staining was already high even in untreated adipocytes and did not change after PA treatment (data not shown). However, we performed double staining for p16^INK4a^ and γH2A.X in order to further support the characterization of the senescent phenotype.

The expression of inflammatory markers (suppl. Fig. 1B) as well as mitochondrial superoxide (MitoSOX, suppl. Fig. 1C) was not changed significantly after FA treatments. However, there was a trend towards increased inflammation (IL-6 and TNF-α) and increased mitochondrial ROS levels after PA treatment, but decreased mitochondrial ROS levels after OA treatment.

### 3.3. Nitrate does not attenuate palmitic acid-induced cellular stress and senescence markers in human adipocytes

**Fig. 3.**
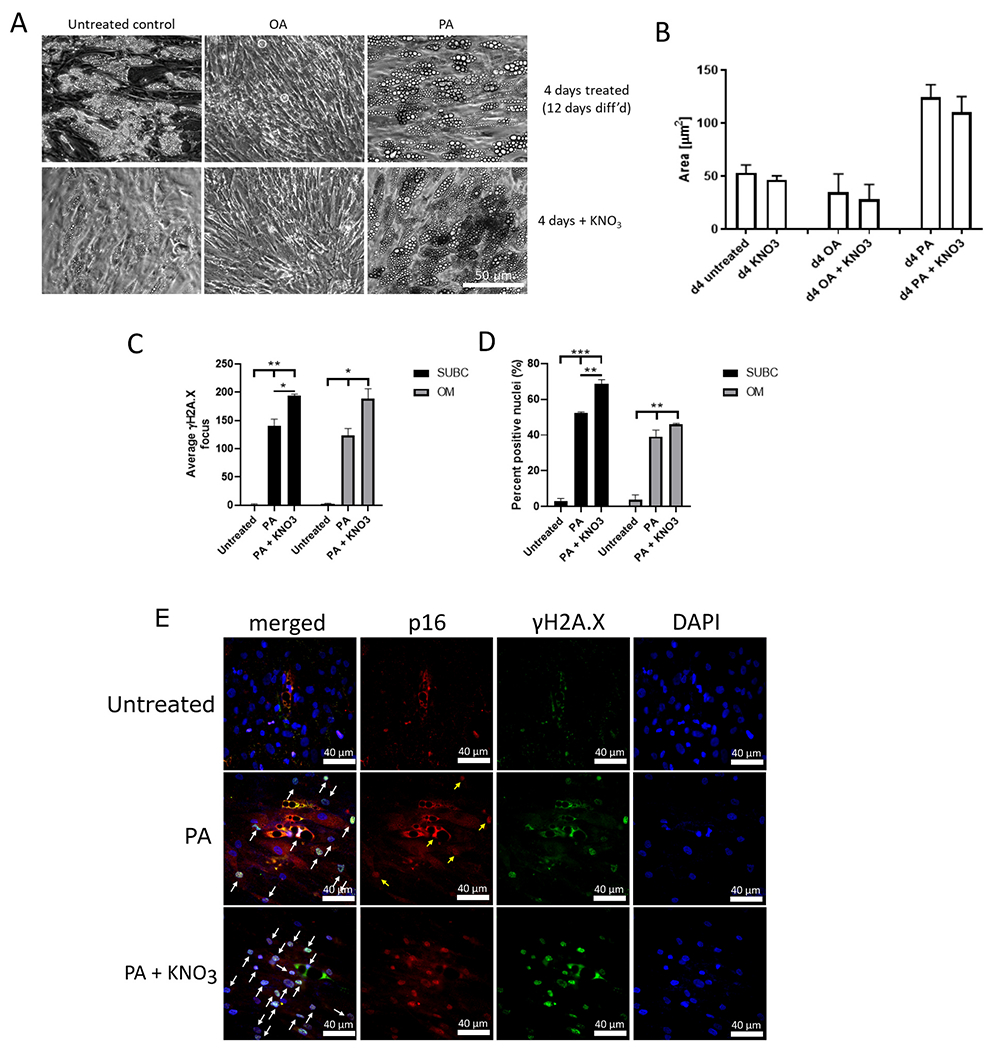
Effect of 2 days 100 μM nitrate treatment on PA-induced LD size, DNA damage, and p16 staining in primary adipocytes. A) Representative images (200× magnification, scale bar is 50 μm) of lipid droplet sizes after 4d FFA and 2 days of nitrate treatment in primary adipocytes (Lonza). B) Change in lipid droplet size in adipocytes, quantified as area (μm^2^). C) Average γH2A.X DNA damage focus count per nucleus after 4 days of 150 nM FFA and 2 days of 100 μM nitrate treatment of donor-derived adipocytes. D) Percentage of p16^INK4a^-positive nuclei quantified by immunofluorescence staining after FFA and nitrate treatment of donor-derived adipocytes. E) Representative IF images for DAPI (blue, right), γH2A.X (green) and p16^INK4a^ (red) as well as merged (left) in untreated and PA- and PA plus nitrate treated adipocytes. Scale bars are 40 μm. Arrows label nuclei positive for both γH2A.X and p16^INK4a^. * p < 0.05, ** p < 0.01, *** p < 0.001 by one-way ANOVA and Holm-Sidak *post hoc* test. n = 3 donors for all treatments. (For interpretation of the references to colour in this figure legend, the reader is referred to the web version of this article.)

There was no significant change in lipid droplet size after nitrate treatment in primary adipocytes (Lonza) (Fig. 3A, B). Nitrate treatment also did not significantly change mean γH2A.X DNA damage focus number or p16^INK4a^ staining between FA-treatment and FA plus nitrate treatment in these cells (data not shown).

Importantly, we were able to reproduce effects of PA on DNA damage and p16^INK4a^ immunofluorescence demonstrated initially on human subcutaneous adipocytes obtained from Lonza in both subcutaneous and omental adipocytes obtained from different primary donors from Mayo Clinic (Fig. 3C, D). However, nitrate treatment not only failed to exert any beneficial effects on these two senescence markers in subcutaneous and omental adipocytes, but rather significantly increased them on top of the damage and p16^INK4a^ signals induced by PA treatment (Fig. 3C, D, E). However, we are not sure about the mechanism for this effect. In addition, we also did not find any beneficial effects of nitrate treatment on the expression of the beiging markers, *UCP1, CPT1a,* and *PGC-1α,* of cultured adipocytes after FFA treatment in primary adipocytes (Lonza) (suppl. Fig. 2). Together, these data suggest that nitrate treatment was unable to induce beiging or alleviate the effects of PA.

### 3.4. OA alleviates DNA damage induction by PA but not the number of lipid droplets or p16^INK4a^ levels

**Fig. 4.**
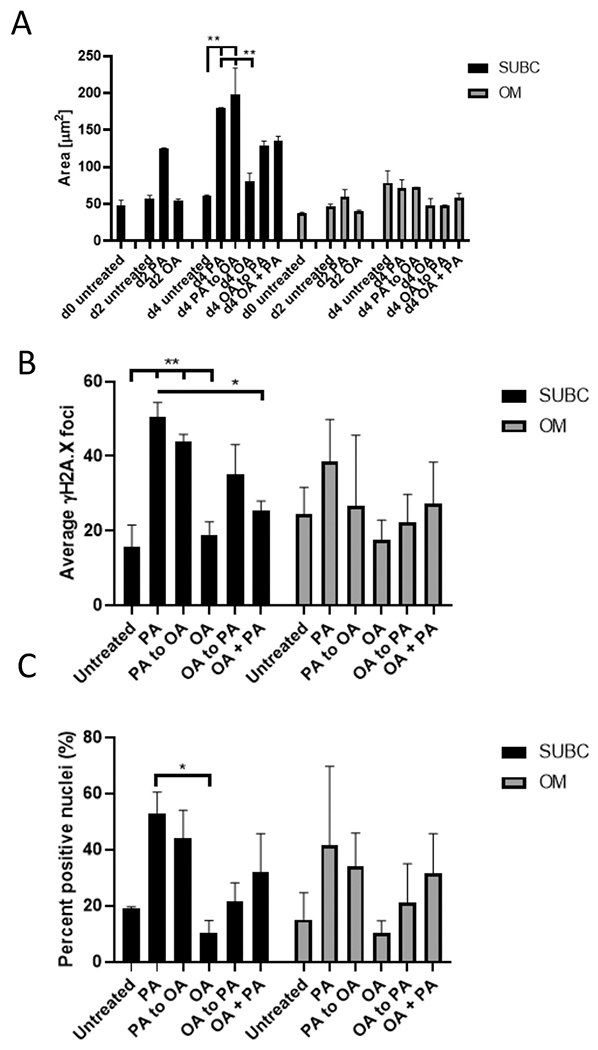
Effect of FFA treatment and co-treatment on donor-derived subcutaneous and omental adipocytes on LD size and senescence markers. A) Lipid accumulation analyzed by day and given as area. B) Quantification of the number of DNA damage foci per nucleus. C) Percentage of p16^INK4a^ -positive nuclei. n = 3 donors for all subcutaneous and omental adipocytes. Comparison between treatments was analyzed by one-way ANOVA with Holm-Sidak *post hoc* test. * p < 0.05 *vs.* indicated, ** p < 0.01 *vs.* indicated.

Based on the strong trend towards a decrease of mitochondrial ROS in the subcutaneous Lonza adipocytes treated with OA (suppl. Fig. 1B), we examined whether OA, instead of nitrate, is able to counteract the negative effects of PA. To achieve this, we supplemented the donor-derived subcutaneous and omental adipocytes with 150 nM OA for 2 days and then with 150 nM PA for 2 days (OA to PA) and *vice versa* (PA to OA) (Fig. 4). We also performed a co-treatment with 150 nM of OA and PA for the entire 4 days of treatment (OA PA). In subcutaneous adipocytes, OA followed by PA treatment produced lipid droplets of 129.04 μm^2^ ± 5.85 which were similar in size to those after 2 days of PA treatment only (Fig. 4A), demonstrating that OA did not prevent lipid accumulation by PA when used consecutively. During FFA treatment of the subcutaneous adipocytes between days 8 and 12, average lipid droplet size of untreated adipocytes (controls) increased from 47.66 μm^2^ ± 7.15 to 57.40 μm^2^ ± 4.10, showing that there was an increase in differentiation-induced lipid accumulation of approximately 10 and 15 μm^2^ over this period. In contrast, PA treatment increased lipid droplet size from 47.66 μm^2^ ± 7.15 to 125.09 μm^2^ ± 0.95 and to 179.36 μm^2^ ± 0.87 in subcutaneous adipocytes after 2 and 4 days, respectively. Thus, there was a total increase in lipid droplet accumulation rate by approximately 9.5-fold due to PA treatment. Similar to Lonza adipocytes, there was no change in lipid accumulation with OA treatment. Likewise, combined OA + PA treatment for 4 days produced similar lipid droplet sizes as 2 days of PA treatment alone, while PA to OA treatment resulted in lipid accumulation levels similar to a full 4 days of PA treatment (Fig. 4A), despite removal of PA after day 2 of treatment. While basal levels of LD were similar in untreated omental adipocytes, neither PA nor any other single or combined treatment generated significant changes in droplet size (Fig. 4A, right side).

OA did not significantly prevent the effect of PA on DNA damage when used before (OA to PA) or after PA (PA to OA) (Fig. 4B). However, subcutaneous adipocytes treated with both OA and PA simultaneously had half the amount of DNA damage of all the other PA single or consecutive treatments (p = 0.003). In principle, PA treatment in omental adipocytes generated around the same range of DNA damage foci (between 20 and 50), but due to high standard deviations in all groups, no significance could be found (Fig. 4B, right side).

As expected, the amount of positively stained nuclei for p16^INK4a^ increased after PA treatment (Fig. 4C). In contrast, p16^INK4a^ immunofluorescence after any consecutive or co-treatments as above did not show significance compared to other groups. Similarly to the results from DNA damage, omental adipocytes showed p16^INK4a^ positive cells (a range of 20–60%) and very extensive heterogeneity, but in general, exhibited trends similar to those in subcutaneous adipocytes (Fig. 4C, right side).

Together, these results suggest that OA is able to prevent the DNA damaging effect of PA when used simultaneously with PA, but not when used before or after PA treatment. In contrast, other parameters, such as p16^INK4a^ staining, did not exhibit a significant effect, but a similar trend to decrease when OA was used.

The effects of OA and PA were less pronounced in omental adipocytes compared to subcutaneous ones, in particular regarding lipid droplet accumulation (Fig. 4A), which could be due to their generally lower differentiation capacity, as described previously (Tchkonia et al., 2005).

## 4. Discussion

Our study examined senescence and inflammatory parameters in primary human adipocytes after treatment with a saturated free fatty acid, palmitic acid. Palmitic acid induced a robust and reproducible increase in lipid droplets during 2–4 days of treatment in subcutaneous adipocytes from different donors. In accordance with previous studies, we found that subcutaneous pre-adipocytes *in vitro* were more predisposed to lipid accumulation than omental pre-adipocytes (Tchkonia et al., 2005; Tchkonia et al., 2002).

Various studies have found that subcutaneous fat accumulates lipids earlier than omental depots (Kotani et al., 1994). Thus, the difference in PA-induced lipid accumulation capabilities between omental and subcutaneous adipocytes in our study most likely reflects these *in vivo* observations. In contrast, in mice, we have previously reported that visceral adipocyte size increased between young (5–10 months) and middle-aged mice (12–20 months) (Ishaq et al., 2018a). In addition, our study of dietary restriction demonstrated that adipocyte size as a readout for lipid accumulation is malleable in visceral adipocytes *in vivo* (Ishaq et al., *2018b*). However, in both *in vivo* studies, these results were not compared to those in subcutaneous WAT. Others have demonstrated that there are differential sensitivities of subcutaneous and visceral depots in rats treated with high or low doses of corticoids (Nunes et al., 2017). At low doses, only subcutaneous fat depots had increased their lipid storage capability and activity of lipid synthesising enzymes while these parameters were increased in visceral fat at high doses (Nunes et al., 2017). Another study confirmed genetic and metabolic differences between subcutaneous and visceral locations at the level of the corresponding adipose-derived stem cells using gene expression analysis (Kim et al., 2016).

Both subcutaneous and visceral adipocytes had comparable amounts of DNA damage and frequency of p16^INK4a^ positive cells as markers of cellular senescence when challenged with PA. Thus, our results do not support the conclusion that hypertrophy of adipocytes is directly linked to a senescence phenotype in general, since while in subcutaneous adipocytes there seems to be an association, we did not find the same in omental adipocytes. Despite a clear increase in the number of p16^INK4a^ positive adipocytes, *p16*^*INK4a*^ gene expression was not upregulated under these conditions. p16^INK4a^ induction during senescence is associated with prevention of G1/S cell cycle transition due to the function of p16^INK4a^ as a cell cycle kinase inhibitor through reinforcement of the p21^CIP1^-induced cell cycle arrest (Rayess et al., 2012). While DNA damage occurs very early during senescence induction, p16^INK4a^ only appears after around 10 days in human fibroblasts (Hewitt et al., 2012). Remarkably, p16^INK4a^ was upregulated much faster in our adipocyte system than in previously published human fibroblast studies: after 4 days of PA treatment compared to 10-days of incubation after irradiation in fibroblasts (Alcorta et al., 1996; Stein et al., 1999). However, the exact kinetics of *p16*^*INK4a*^ up-regulation after various stressors resulting in senescence is not well understood and involves a complex transcriptional regulation at the epigenetic level and including multiple transcription factors. Finally, there was a far larger increase in telomere-associated foci after PA treatment (Fig. 2C) compared to general DNA damage (Fig. 2D). This result confirms the increased sensitivity of telomeres to DNA damage, as shown by us and others previously (Hewitt et al., 2012; Petersen et al., 1998; von Zglinicki et al., 2000). Interestingly, there was a good agreement in the magnitude of increase between the amount of p16^INK4a^ positive cells and the amount of general DNA damage. In order to strengthen the characterization of a senescent phenotype, we performed double-staining for γH2A.X and p16^INK4a^ (Fig. 2G). Together with telomeric damage (shown here as TAFs) and the SASP, these two markers are considered to be robust and reliable markers for senescence in postmitotic tissues (von Zglinicki et al., 2021), including in white adipocytes (Smith et al., 2005). Surprisingly, we did not find a major increase in inflammatory response due to PA treatment and oxidative stress was not increased significantly either.

In most reports, the damaging effects of PA have been described as cytotoxic through effects on the endoplasmic reticulum (Robblee et al., 2016; Shen et al., 2017; Guo et al., 2007; Sun et al., 2015), while we describe here that PA induces DNA damage and senescence in accordance with previous reports (Beeharry et al., 2003). Since differentiated adipocytes are postmitotic cells, the senescence phenotype, excluding the occurrence of a cell cycle arrest, is slightly different compared to dividing cells that become senescent. However, SASP factors, such as IL-6, have been identified at the protein level in other postmitotic cells, such as Purkinje neurons in brain tissue from old mice (Jurk et al., 2012). We also demonstrated previously an increase in some inflammatory markers such as TNF-α in perirenal WAT from very old (30 months) mice, which could be caused by increased amounts of macrophages in the tissue (Ishaq et al., 2018a). Since these macrophages are not present in adipocyte cultures, we suggest that increased inflammation requires presence of such inflammatory cells to promote an inflammatory phenotype after exposure to senescence-inducing stress. The lack of many SASP factors from isolated, cultured cells compared to (heart) tissue has also been described in the case of cardiomyocytes (Anderson et al., 2019). The authors suggested that in heart, connective tissue fibroblasts may play an essential role as mediators of senescence.

Our study did not demonstrate any beneficial effect of inorganic nitrate treatment for alleviating the senescent phenotype or adipocyte hypertrophy. We also did not find evidence for a nitrate-related beiging effect in the “after”-differentiation nitrate treatments. In contrast, Roberts and colleagues found that nitrate induced beiging in rodent adipocytes during their *in vitro* differentiation (“during differentiation”) (Roberts et al., 2015). A possible reason for this discrepancy could be species differences since we used primary human adipocytes. Beiging involves a shift in fat metabolism and mitochondrial energy generation to a more uncoupled phenotype. It is thought that this includes a switch in gene expression during adipocyte differentiation from a white adipocyte type to a more brown one. In addition, it has also been shown that interleukin IL-6 is able to induce a beiging/ browning effect in adipocytes (Li et al., 1868).

While nitrate treatment did not alleviate induction of a senescent phenotype after PA treatment and even seemed to exacerbate the damaging and senescence-associated phenotype in cultured adipocytes, we found that OA treatment together with PA treatment had a beneficial effect. It decreased DNA damage as a senescence parameter, while not changing p16^INK4a^ levels or lipid droplet size significantly in our rather limited sample size. Our result about DNA damage is similar to that obtained by Beeharry and colleagues using linoleic acid (LA) and PA together, where the former was able to reduce DNA damage caused by the latter in human primary fibroblasts and rat islet-tumor derived cells (Beeharry et al., 2003). The authors interpreted their data as being related to the antioxidant and anti-apoptotic capacity of LA.

It has been shown previously that the effect of OA on lipid esterification differs across tissues (Ducheix et al., 2017; Burhans et al., 2015; Kwon et al., 2014). For example, OA did not promote lipid accumulation in our human adipocyte cell system, unlike in mice, in which OA was associated with increased hepatic lipogenesis due to liver-specific receptors (Burhans et al., 2015). Pre-treatment of mouse neuroblastoma cells *in vitro* with OA can also protect against PA-induced apoptosis, inflammation, and insulin resistance (Kwon et al., 2014). In our adipocyte system, at the same concentration as PA, OA protected against the effects of DNA damage-inducing effects of PA when used simultaneously. This corresponds well to previous data on skeletal muscle cells where OA treatment prevented mtDNA damage and prevented PA-induced one when OA was applied together with PA (Yuzefovych et al., 2010). Translated into human nutrition, our result of a beneficial effect of OA as opposed to PA supports the benefits of a Mediterranean diet, which is rich in olive oil and oleic acid. Thus, consuming food products rich in oleic acid could lead to a more balanced and healthier diet and help to prevent obesity and related diseases.

However, in order to understand the underlying signaling pathways, further studies into the molecular mechanisms of how PA and OA modulate senescence are required. In particular, the occurrence of DNA damage despite a lack of measurable oxidative stress in our study raises questions regarding the underlying mechanisms. Lee and colleagues recently demonstrated that senescence induction by PA in endothelial cells works through stimulation of autophagy (Lee et al., 2021) while Hewitt and co-authors have recently demonstrated that accumulation of the autophagy receptor protein p62 associated with suppression of autophagy promotes DNA damage due to decreased DNA repair (Hewitt et al., 2016). It was also demonstrated that PA induces mitochondrial damage and release of mitochondrial DNA into the cytosol activating the cGas-STING pathway in the same cell type (Yuan et al., 2017). Interestingly, STING needs to be palmitoylated for activity (Mukai et al., 2016) and resides within the endoplasmatic reticulum (ER) and on ER-mitochondria-associated membranes (Smith, 2021), while PA is known to exert ER stress (Yin et al., 2015). STING has been shown to be upregulated after PA treatment (Hu et al., 2020) and can induce autophagy that is compromised due to PA. Thus, there are a number of interconnected signaling pathways involved in the damaging phenotype of PA that warrant further investigation.

## 5. Conclusions

The results of the current *in vitro* study using differentiated human primary adipocytes suggest that treatment with saturated fatty acids such as palmitic acid results in higher lipid droplet content (a marker for hypertrophy), increased DNA damage (generally and even more pronounced at telomeres), as well as the occurrence of p16^INK4a^ positive nuclei. All these parameters are indicative of the development of senescence in connection with obesity after consumption of saturated fat in our food. However, differences in the amount of senescence induction between subcutaneous and visceral/omental adipocytes also demonstrate that lipid hypertrophy can be associated with senescence markers, but is not necessarily causative for them. Our results suggest a tight relationship between obesity and senescence in fat cells and emphasize the requirement for healthy food in order to mitigate obesity and to alleviate detrimental effects of the ageing process that are known to be associated tightly with nutrition.

## Supplementary Material

supplementary

## Figures and Tables

**Table 1:** Primer sequences used in qPCR. Annealing temperatures for all primer pairs were 60 °C. TNF-α - Tumor necrosis factor α, IL - interleukin, UCP1 - uncoupling protein 1, CPT1 - carnitine palmitoyltransferase 1, PGC1-α -peroxisome proliferator γ coactivator 1-α.

Primer name	Forward primer 5′- > 3′	Reverse primer 5′- > 3′
18S	CCAAGATCCAACTACGAGCTT	GGCCCTGTAATTGGAATGAGTC
p16^INK4a^	GGGTCGGGTAGAGGAGGTG	GCCTCCGACCGTAACTATTCG
p21^Cip1^	TATGGGGCTGGGAGTAGTTG	ACATTTGGAGAGCTCCCGTC
TNF-α	CCCAGGGACCTCTCTCTAATC	ATGGGCTACAGGCTTGTCACT
IL-6	GTGCCTCTTTGCTGCTTTCAC	GGTACATCCTCGACGGCATCT
Il-1β	AAACAGATGAAGTGCTCCTTCCAGG	TGGAGAACACCACTTGTTGCTCCA
UCP1	CCGGCTCCAGGTCCAAG	CCGATCCTGAGAGAGGCG
CPT1a	GAGAGGAGACAGACACCATCCA	CTGAGGATCCGAGGTATTGTCC
PGC1-α	CTCTGGAACTGCAGGCCTAACT	TGGAGTTATTGCCTTGTGTACCAG

## Data Availability

Data will be made available on request.
