# Supplementary material for: Palmitate induces DNA damage and senescence in human adipocytes in vitro that can be alleviated by oleic acid but not inorganic nitrate
Source: Exp Gerontol. Author manuscript; Available in PMC 2022 Jun 22. (PMC9214712; doi:10.1016/j.exger.2022.111798)
Supplement: supplementary [file NIHMS1814755-supplement-supplementary.pdf]

The following are the supplementary data related to this article.

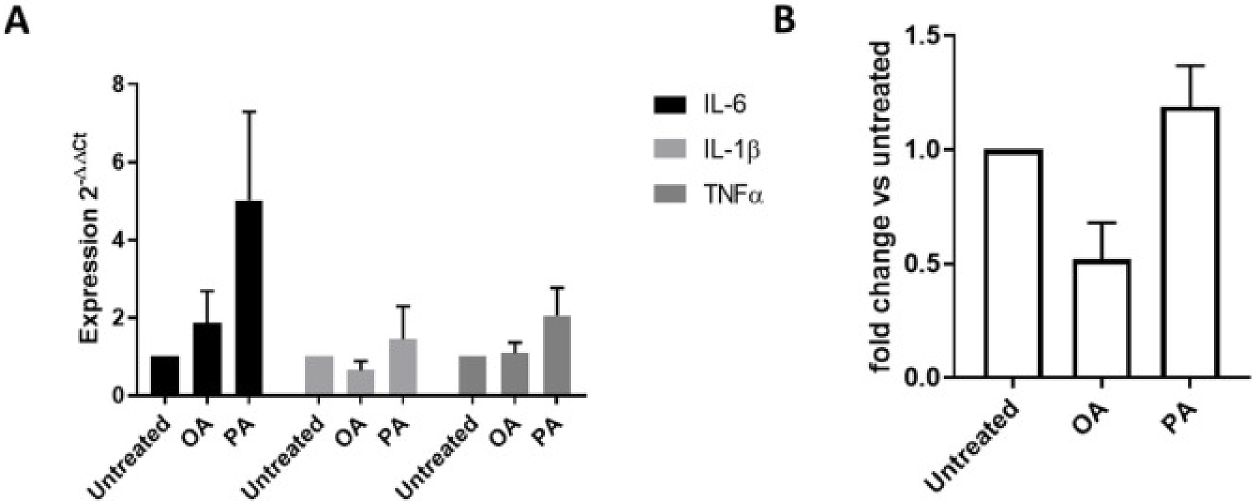

1. Download : Download high-res image (139KB)
2. Download : Download full-size image

Supplementary Fig. 1. Effect of 4 days 150 nM FFA treatment on senescence and inflammation markers as well as ROS levels in human primary subcutaneous adipocytes (Lonza). *p16*^{INK4a} and *p21*^{CIP1} and B) the inflammation markers, *IL-6*, *IL-1β*, and *TNFα*. C) Mitochondrial superoxide measurement by flow cytometry using a mitosox probe; n = 4 independent experiments.

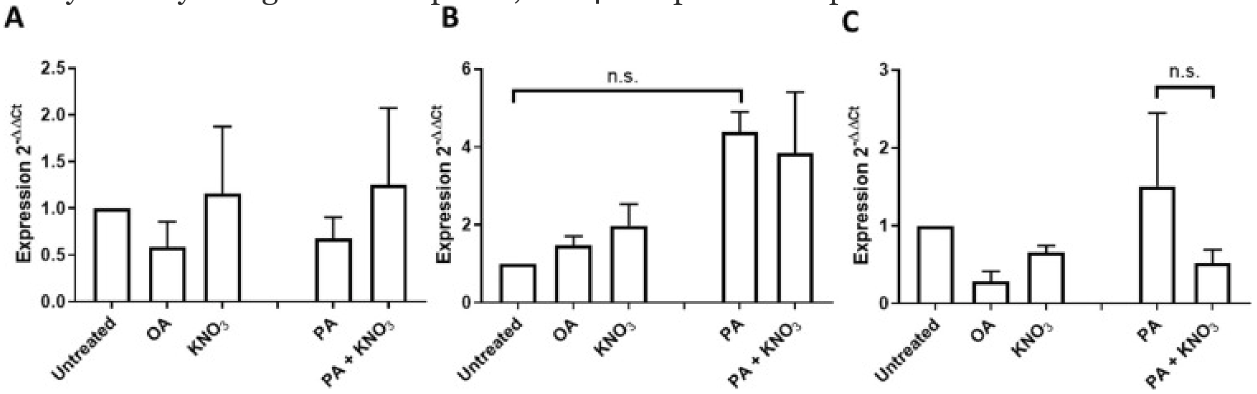

1. Download : Download high-res image (150KB)
2. Download : Download full-size image

Supplementary Fig. 2. Effect of 2 days treatment with 100 µM nitrate on beiging markers "after differentiation" treatment of human subcutaneous adipocytes (Lonza). Expression of beiging markers A) UCP1, B) *CPT1a*, and C) *PGC-1α*.
